# Evidence of oxidative stress-induced senescence in mature, post-mature and pathological human placentas

**DOI:** 10.1016/j.placenta.2018.06.307

**Published:** 2018-08

**Authors:** Tereza Cindrova-Davies, Norah M.E. Fogarty, Carolyn J.P. Jones, John Kingdom, Graham J. Burton

**Affiliations:** a Centre for Trophoblast Research, University of Cambridge, UK; b Francis Crick Institute, London, UK; c Maternal and Fetal Health Research Centre Division of Developmental Biology & Medicine School of Medical Sciences Faculty of Biology, Medicine and Health University of Manchester, Manchester Academic Health Science Centre, St Mary's Hospital Oxford Road Manchester M13 9 WL, UK; d Mount Sinai Hospital, University of Toronto, Canada

**Keywords:** Senescence, Syncytiotrophoblast, Oxidative stress

## Abstract

**Introduction:**

Premature ageing has been implicated in placental dysfunction. Senescence can be activated by oxidative stress, a key intermediary in the pathophysiology of pre-eclampsia. We examined senescence markers across normal gestation, and in pathological and post-mature pregnancies. Inducers of oxidative stress were used to mimic senescence changes in term explants.

**Methods:**

Placental samples were collected with ethical approval and informed consent: first and second trimester samples from surgical terminations; term and pre-term controls, and early-onset pre-eclampsia samples from caesarean deliveries. Paraffin and EM blocks of post-mature placentas were from an archival collection. Term explants were subjected to hypoxia-reoxygenation (HR) or hydrogen peroxide (H_2_O_2_).

**Results:**

p21 was increased significantly in term homogenates compared to first and second trimester samples, and was significantly higher in PE compared to term controls. Immunostaining revealed nuclear localisation of p21 and phosphorylated histone γH2AX in syncytiotrophoblast, with abundant foci in pathological and post-mature placentas. Abnormal nuclear appearances were observed in post-mature placentas. Sudan-Black-B staining demonstrated abundant lipofuscin, an aggregate of oxidised proteins, lipids and metals, in post-mature and pathological placentas. The percentage of nuclei positive for 8-hydroxy-2′-deoxy-guanosine, a marker of oxidised DNA/RNA, was increased in pathological placentas compared to age-matched controls. These changes could be mimicked by challenge with HR or H_2_O_2_.

**Discussion:**

Senescence markers increase in normal placentas with gestational age, and are exaggerated in post-mature and pathological cases. Oxidative stress triggers equivalent changes in explants, and may precipitate senescence *in vivo*. The consequent pro-inflammatory senescence-associated secretory phenotype may contribute to the pathophysiology of pre-eclampsia.

## Highlights

• Markers of senescence increase in healthy placentas with advancing gestational age.
• Senescence changes are most extensive post-term, and in early-onset pre-eclampsia.
• Oxidative stress can induce placental senescence *in vitro*.
• Senescence changes are primarily restricted to the syncytiotrophoblast.

## Introduction

Senescence is a critical feature of mammalian cells, and can be both beneficial and detrimental. Thus, oncogene-triggered senescence suppresses tumour growth [1], whereas loss of tissue homeostasis during ageing [2,3] contributes to atherosclerosis [4] and neurodegeneration [5]. Senescence has been implicated in cell fusion [6], and so is relevant to the syncytiotrophoblast of the human placenta. Transfection of normal and malignant non-trophoblast cells with syncytin 1 causes cellular fusion and formation of syncytia, which exhibit features of senescence. Replicative senescence is a permanent cell cycle arrest resulting from high levels of the cyclin kinase inhibitors p21/or p16. Cells remain metabolically active and adopt characteristic phenotypic changes [7]. Cell senescence can also be activated by a variety of intrinsic and extrinsic stresses, including oxidative stress, DNA damage, nucleolar stress, epigenetic stress, telomere damage, chronic mitogen signaling, and oncogene activation/inactivation. For example, reactive oxygen species activate DNA damage response (DDR) by perturbing gene transcription and DNA replication, and by inducing telomere shortening. Senescence stressors ultimately activate the p53 and/or p16^Ink4a^ pathways; p53 activation inducing p21 and cell cycle arrest via cyclin E-Cdk2, while p16^Ink4a^ targets cyclin D-Cdk4 and D-Cdk6 complexes [8]. These actions prevent inactivation of RB (retinoblastoma protein), leading to continued repression of E2F target genes required for S-phase onset. Some cells form regions of highly condensed chromatin, called senescence-associated heterochromatin foci, that sequester genes facilitating cell-cycle control. These foci contain chromatin modifications, such as activated H2AX (γH2AX) and H3K9me, and reinforce senescence-associated growth arrest [9,10]. One emerging feature of senescent cells is inflammation. Senescent cells secrete a unique cocktail of factors, collectively known as senescence-associated secretory phenotype (SASP), which includes pro-inflammatory cytokines and chemokines, and various growth factors and proteases that together alter the tissue microenvironment [11,12]. Damaged human cells develop persistent chromatin lesions bearing hallmarks of DNA double-strand breaks, which initiate increased secretion of inflammatory cytokines. Evidence suggests that a feedback loop involving mitochondrial dysfunction and ROS production might be important in various physiologically relevant forms of cell senescence [13]. Senescent cells have distinct phenotypic features, including flattened and enlarged morphology with marked actin stress fibres, stability in culture, and increased senescence-associated β-galactosidase (SA-β-gal) activity [14]. SA-β-gal is the most reliable senescence biomarker. However, the SA-β-gal activity can be detected only in snap-frozen tissues, not in archival paraffin-embedded sections. A recent study validated the histochemical Sudan-Black-B (SBB) specific stain of lipofuscin, an aggregate of oxidised proteins, lipids and metals known to accumulate in aged tissues, as an additional reliable approach to detect senescent cells independently of sample preparation [15]. In addition, cytoplasmic chromatin fragments can pinch off from intact nuclei of primary cells during senescence. Recent evidence shows that the presence of DNA in the cytoplasm can initiate a SASP response by activating the innate immunity cytosolic DNA-sensing cyclic GMP-AMP synthase (cGAS)-STING pathway [16,17]. The enzyme cyclic GMP-AMP synthase (cGAS) acts as a first responder as it binds to cytoplasmic DNA, and catalyses production of the molecule cGAMP and triggers a pro-inflammatory response [16,17].

Placental dysfunction is the main cause of many placental-related pathologies. Recent studies indicate that premature placental ageing might be involved in this process. Placental ageing was first described in the 1970s in animal models [[18], [19], [20]] when it was suggested that the rapidly proliferating placental cells start to age as the placenta reaches term. This physiological ageing has been documented in a recent study of human term placentas (39 wk), late term placentas (>41wk) and placentas from unexplained stillbirth. Increased aldehyde oxidase 1 expression, increased oxidation of DNA/RNA (8-OHdG) and lipid, perinuclear location of lysosomes, and larger autophagosomes were observed in both later term and stillborn placentas, compared to placentas from term deliveries [21]. In addition, the significant increase in the rate of stillbirth commencing at 36–37 wk gestation, suggests that placental ageing might increase the risk of fetal demise [22,23]. Thus the first aim of this study was to examine evidence of senescence across normal gestation, and to evaluate senescent changes in an archival collection of post-mature placentas (delivered 7–20 after the due date).

Strong evidence exists that placental oxidative stress is a key intermediary event in the pathology of preeclampsia (PE) and intrauterine growth restriction (IUGR) [[24], [25], [26]], secondary to deficient conversion of the uterine spiral arteries and malperfusion [27]. Damage to DNA and proteins can result in shortened telomeres in the trophoblast in these pregnancies [28], and induce senescence. In addition, a recent microarray analysis demonstrated increased expression of *p21*, *p53*, *APE1* and *IL-6* in PE and IUGR placentas [29]. Therefore, the second aim of this study was to investigate senescent changes in pathological pre-eclamptic and IUGR pregnancies, and to use inducers of oxidative stress to test whether they are capable of recapitulating senescence changes in healthy term placental explants *in vitro*.

## Methods

### Tissue collection - first, second trimester and term tissue

All material was collected with informed written patient consent. First and second trimester placental samples were collected from surgical terminations with approval of the Joint UCL/UCLH Committees on the Ethics of Human Research (05/Q0505/82). The samples were collected using a chorionic villus sampling-like (CVS) technique under ultrasound guidance from the central region of the placenta. Gestational age was estimated from the crown rump length of the fetus. Part of the sample was frozen immediately (<2 min) in liquid nitrogen (time zero, T_0_), and part was fixed in 4% paraformaldehyde (PFA) and embedded in paraffin wax for immunohistochemistry (IHC). Frozen samples of first trimester (7–8 wk; N = 5) and second trimester (13–17 wk; N = 6) placentas were analysed in this study. To study changes across gestation, samples were collected from uncomplicated singleton pregnancies at term (N = 5; 39 weeks) with approval from the Cambridge Local Ethics Committee and with informed written patient consent immediately after delivery by elective caesarean section (tissues were harvested within 10 min of delivery). The most common indication for caesarean section was a history of previous section. Samples were transported to the laboratory on ice for further processing or for use as explant cultures (see below).

### Tissue collection – post-mature placentas and their term controls

Paraffin and EM blocks of term healthy placentas (N = 5) and post-mature placentas (delivered 7–20 d after due date, N = 6) were from an archival collection from the University of Manchester [30].

### Tissue collection – pathological samples and appropriate controls

Pathological placentas and respective term and preterm controls were collected with ethical approval from the Mount Sinai Hospital, Toronto, Canada, following caesarean delivery. Samples included term controls (N = 7, 39 wk), pre-term controls (N = 6, 29 wk), early-onset PE (N = 10, 30 wk) and normotensive IUGR placentas (N = 6, 31.5 wk) from a cohort of samples previously included in our publications [31,32]. The selection criteria used for pre-eclampsia was the onset of new hypertension and proteinuria after 20 weeks of gestation; hypertension being defined as two or more recordings of a diastolic blood pressure of 90 mmHg or more taken at least 4 h apart, proteinuria taken as the excretion of 300 mg protein or more over a 24 h period. All pre-eclampsia cases were early-onset, defined as an onset between 20 and 34 weeks of gestation, and all were associated with IUGR. IUGR was defined according to established criteria [33], i.e. fetal biometry was below the 10th centile for gestational age according to local reference values, and umbilical artery Doppler flow velocity was abnormal (Doppler score class II and III). The pre-term control placentas were from women who had preterm but otherwise uneventful pregnancies, or late terminations of pregnancy for medical reasons. Fetal growth had been normal and birthweight was always above 10th percentile. The umbilical and uterine Doppler measurements were normal. The membranes were never ruptured for longer than 12 h and signs of chorioamnionitis were also excluded by histological studies. The cases included in this study all delivered by a caesarean section, and comprised of 2 late terminations of pregnancy (due to autosomal recessive polycystic kidneys or renal agenesis), 3 cases of early pre-mature rupture of membranes (no chorioamnionitis), and 1 case of early pre-term labour. However, preterm deliveries by definition are not normal controls. We therefore included an additional group of term controls from healthy uneventful pregnancies with normal umbilical and uterine artery Doppler waveforms. Placental tissue samples were snap-frozen immediately and stored at −80 °C, and fixed in 4% PFA and processed for IHC. All placental samples were obtained from 3 to 4 placental regions, and several regions were incorporated into paraffin sections of the pathological placentas. However, these different samples were not pooled for Western blotting, for which a piece was cut from a block of frozen tissue.

### Culture of placental explants

Villous samples were taken midway between the chorionic and basal plates, from the periphery of lobules free of visible infarction, calcification, haematoma or tears from term non-laboured placentas within 10 min of delivery, as previously described [34]. Following transport and tissue dissection, small placental explants (∼5 mm thick) were cultured under following conditions for 24 h: normoxia (10% O_2_, 5% CO_2_), hypoxia-reoxygenation (HR; hypoxia (0.5% O_2_/94.5% N_2_/5% CO_2_) for 1 h, subsequent reoxygenation at normoxia (10% O_2_/85% N_2_/5% CO_2_) for 24 h), or H_2_O_2_ (1–1000 mM for 24–48 h).

### Western blotting

We followed our previously described protocol [31]. Briefly, frozen tissue samples were homogenised in ice-cold lysis buffer and protein concentrations determined using a BCA protein assay kit (Sigma, Poole, UK). Lysates were mixed with 3 x SDS PAGE sample buffer, boiled for 5 min and allowed to cool to room temperature. Equal amounts of protein (30–50 μg) were separated by sodium dodecyl sulphate-polyacrylamide gel electrophoresis, using 7.5–12.5% polyacrylamide resolving gels, and transferred onto nitrocellulose membrane (Invitrogen, Paisley, UK), and subjected to immunoblot analysis. Membranes were blocked for 1 h at 25 °C in 5% milk diluted in Tris-buffered saline (TBS) and 0.1% Tween 20 and incubated with the following primary antibodies overnight at 4 °C: anti-p21 (Cell Signaling; #2947), anti-p16 (Abcam; ab51243), or anti-cGAMP (Abcam; ab48508). After washing and incubating with secondary antibodies, immunoreactive proteins were visualized by the ECL plus chemiluminescence system following the manufacturer's instructions (Amersham Biosciences, Bucks., UK). Protein bands were quantified using Image J software (National Institutes of Health, http://rsb.info.nih.gov/ij/). Protein loading was normalized against Poncaeu S staining [35]. The values are expressed as a percentage of the control lysate (100%) for each experiment.

### Immunostaining

Immunohistochemistry was performed as previously described [31,36]. Briefly, following rehydration in xylene and graded ethanol, endogenous peroxidase was quenched with H_2_O_2_ and sections underwent heat antigen retrieval in Tris-EDTA buffer. The following antibodies were applied and incubated at 4 ֯C overnight: p21 (Cell Signaling; #2947), p16 (Abcam; ab51243), cGAMP (Abcam; ab224144), γH2AX (Cell Signaling; #7631) and 8-hydroxy-2′-deoxy-guanosine (8-OHdG; Abcam; ab48508). The next day, sections were incubated with biotin-labelled species specific antibodies, Vectastain Elite ABC kit (Vector Labs) and SigmaFast DAB (Sigma, Poole, UK).

Stained slides were scanned using a NanoZoomer scanner (Hamamatsu, Welwyn Garden City, UK). Each slide was visualized and several images (6–10 per placenta) were captured per placenta, with the observer blind to the study group. The number of p21 and 8OHdG positive trophoblast nuclei was quantified and expressed as a percentage of the total number of trophoblast nuclei counted. The staining of cGAMP expression in the trophoblast was determined semi-quantitatively using Image J.

### Sudan-black-B staining for lysosomes

Placental paraffin sections were dewaxed and brought to 70% ethanol. They were incubated in a saturated Sudan-Black-B solution (dissolved in 70% ethanol, freshly filtered before use) by adding a drop of SBB to a clean slide and inverting the section face down in order to prevent dye precipitation for about 8 min. Sections were rinsed in 50% ethanol and placed in distilled water. Nuclear Fast Red stain (0.1% solution, Sigma, UK) was subsequently applied for 2 min, sections washed and mounted in a gelatin/glycerol/phenol aqueous mounting solution. Stained slides were scanned using a NanoZoomer scanner (Hamamatsu, Welwyn Garden City, UK). Each slide was visualized and several images were captured.

### Statistical analysis

Data are expressed as mean ± SD. Comparisons were made using a two-tailed Student's *t*-test where only two groups of samples were compared, or ANOVA with a Tukey's multiple comparison post-hoc test if more than two groups of samples were compared. Differences were considered to be significant at *P* ≤ 0.05.

## Results

### Expression of senescence markers across gestation and in post-mature placentas

**Fig. 1:**
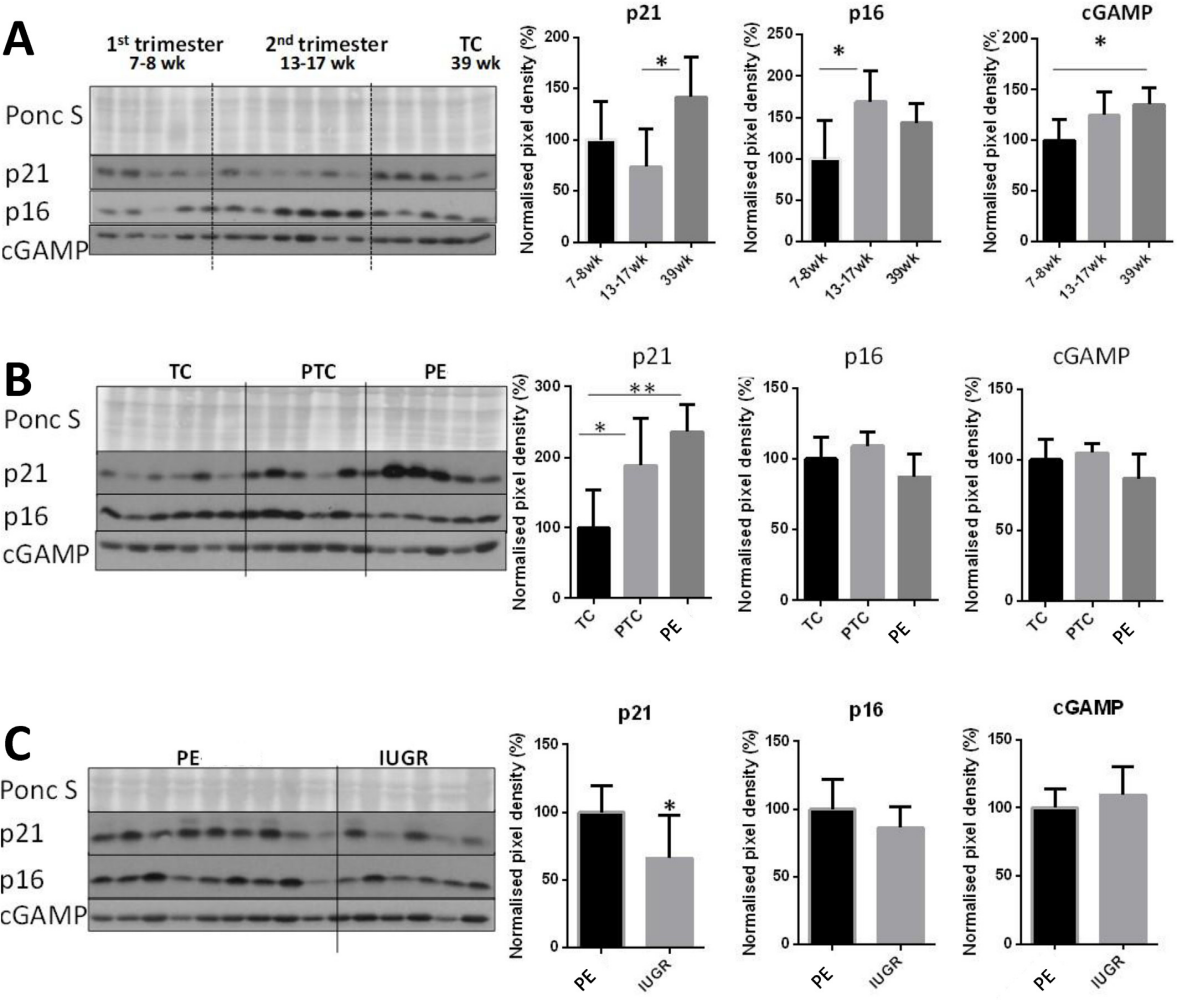
Evidence of senescence across gestation in first, second trimester and term samples collected by chorionic villus sampling technique (A: first trimester (7–8 wk; N = 5), second trimester (13–17 wk; N = 6), term (TC; 39 wk; N = 5)), in pathological placentas (B: term (N = 6, 39 wk) and pre-term controls (N = 5, 29 wk), and early-onset PE (N = 6, 30 wk); C: early-onset PE (N = 9, 30 wk), and IUGR placentas (N = 6, 31.5 wk)) obtained from elective caesarean deliveries. Placental tissue homogenates were probed with anti-p21, anti-p16, or anti-cGAMP antibodies and the signal was quantified. A) Quantification of Western blots revealed significant increase in p21 protein in term placental homogenates compared to first and second trimester samples, while p16 increased significantly in the second trimester but showed no further difference at term. B) The p21 and p16 expression was compared in term and preterm control placentas with samples from early-onset PE (PE). Levels of p21 were lowest in term control placentas, and significantly higher in both preterm controls and PE. There were no significant differences in p16 protein among the three groups. C) Levels of p21 were significantly higher in placentas from PE, and again there was no significant difference in p16. Data are presented as mean ± SD. *p < 0.05. Comparisons were made using ANOVA with a Tukey's multiple comparison post-hoc test in A and B, or using a two-tailed Student's *t*-test where only two groups of samples were compared (C).

**Fig. 2:**
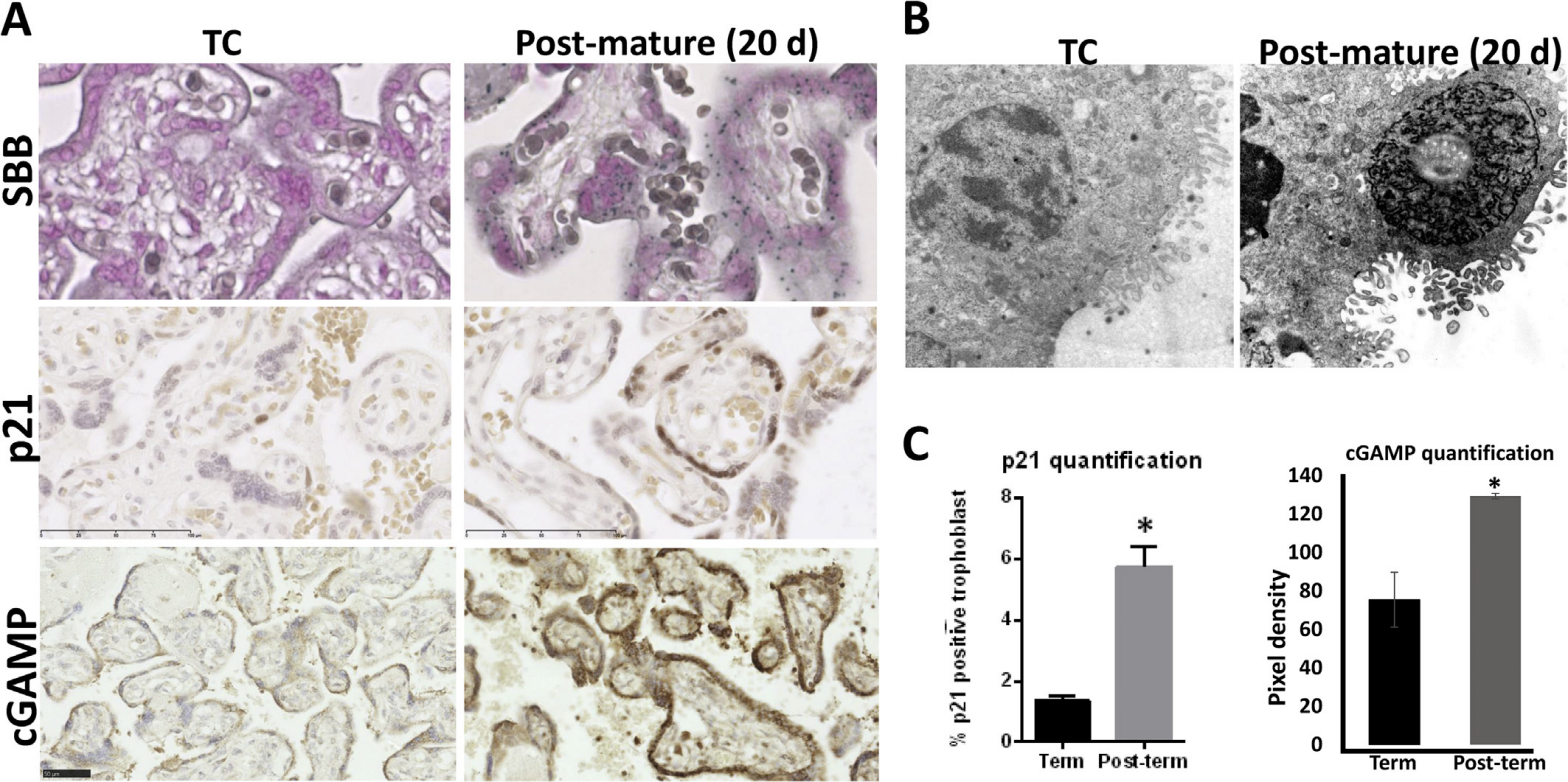
Evidence of senescence in post-mature placentas. Archival sections from term healthy placentas (N = 5) and post-mature placentas (delivered 7–20 d after due date, N = 6) were stained with Sudan-Black-B (SBB) to detect lipofuscin, which is an aggregate of oxidised proteins, lipids and metals, known to accumulate in aged tissues (A), with p21 (A, C) or cGAMP (A, C). Compared to term controls, which only showed sporadic SBB staining, the post-mature placentas showed abundant cytoplasmic staining with SBB (A). B) Archival EM blocks of control and post-mature placentas were examined. Images show dense heterochromatin in a placenta that was delivered 20 days after the due delivery date. C) The number of p21 positive trophoblast nuclei was quantified and expressed as a percentage of the total number of trophoblast nuclei counted (total number of trophoblast nuclei was counted in five fields of view per sample at ×20 magnification, using Image J). Similarly, the staining intensity of the cytoplasmic cGAMP staining was quantified in term and post-term placentas using Image J. * Significant differences (P < 0.05, *t*-test).

Significant increases in p21, p16 and cGAMP were observed in homogenates of healthy placentas with advancing gestational age (Fig. 1A), consistent with a report showing p16, p21, p53 and SA-β-gal in the syncytiotrophoblast at term [6]. Given the rise in p21 with gestational age, we quantified the number of p21-positive syncytiotrophoblast nuclei in an archival collection of post-mature placentas delivered 7–20 days following the due date [30], and observed a significant increase compared to term controls (5.7 vs. 1.3%; Fig. 2A, C). Post-mature placentas also showed abundant staining with the senescence biomarker Sudan-Black-B, which localises to lipofuscin, an aggregate of oxidised proteins, lipids and metals (Fig. 2A). In addition, cGAMP was significantly increased in post-mature placentas, indicating increased cytoplasmic DNA in the syncytiotrophoblast (Fig. 2A, C). Abnormal nuclear appearances were observed in archival EM blocks of post-mature placentas, with dense heterochromatin being observed in some nuclear profiles but dissolution of the chromatin structure in others (Fig. 2B).

**Fig. 3:**
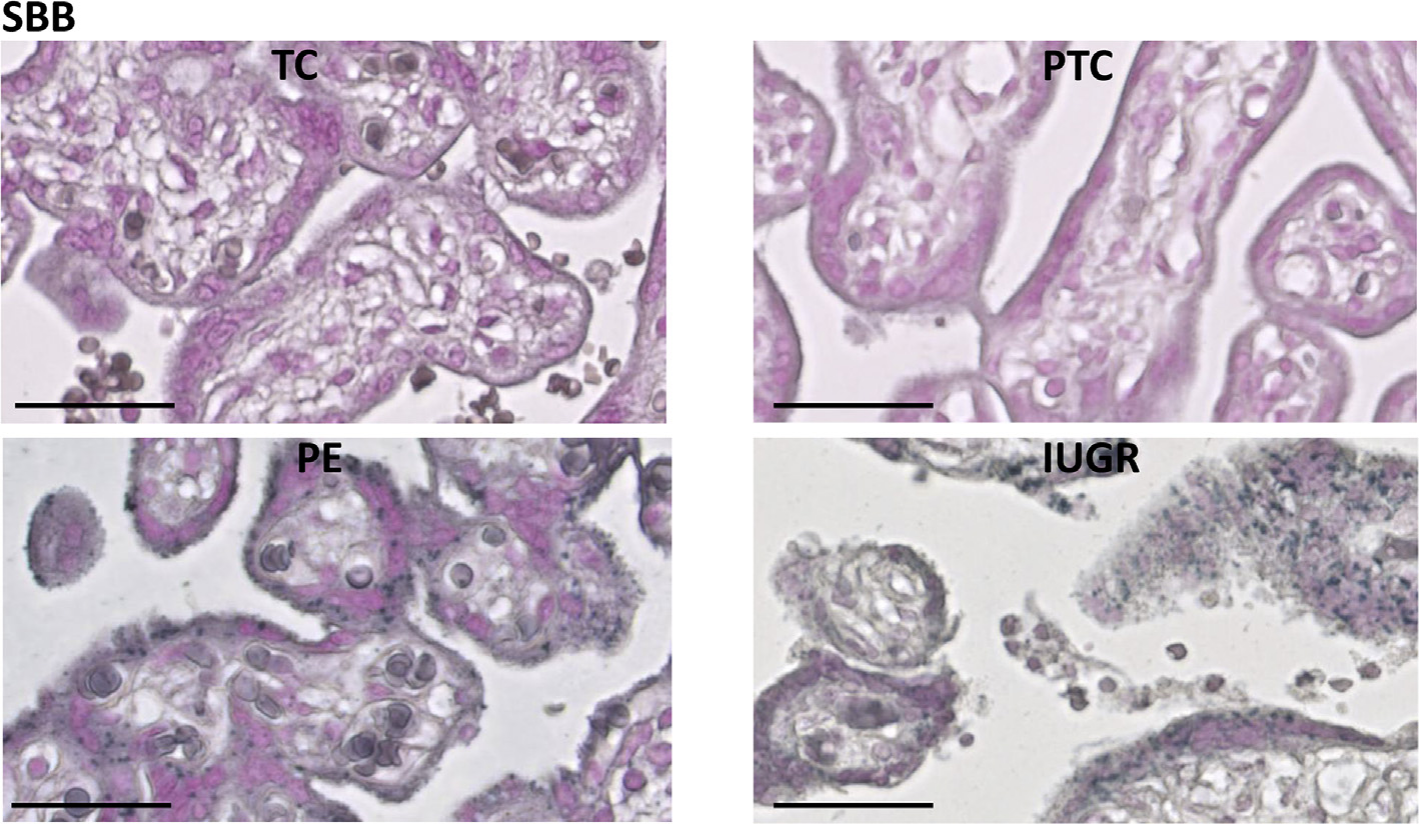
Staining with Sudan-Black-B (SBB) to detect lipofuscin, an aggregate of oxidised proteins, lipids and metals, in control and pathological placentas. Immunostaining revealed abundant nuclear localisation in the syncytiotrophoblast of the pathological placentas.

### Evidence of senescence in pathological placentas

We compared p21, p16 and cGAMP in term and preterm control placentas with samples from early-onset PE (PE). Levels of p21 were lowest in term control placentas, and significantly higher in both preterm controls and PE (Fig. 1B). The increase in preterm controls may reflect the underlying placental pathology prompting their delivery. There were no significant differences in p16 or cGAMP protein among the groups (Fig. 1B).

**Fig. 4:**
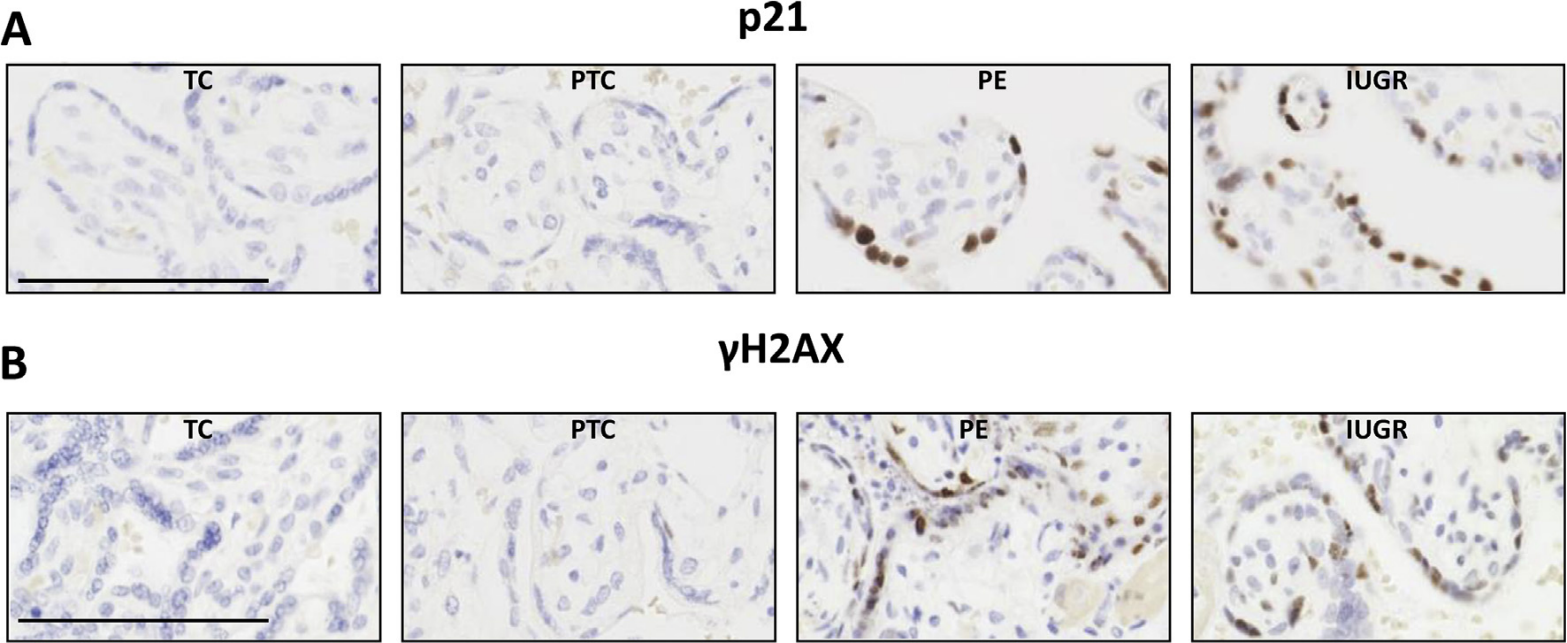
Immunolocalisation of p21 (A) and modified histone, γH2AX (B), in control and pathological placentas. Immunostaining revealed abundant nuclear localisation of p21 (A) and modified histone γH2AX (B) in the syncytiotrophoblast of the pathological placentas.

Placentas from cases of PE suffer more severe malperfusion than those from IUGR alone [37], which may stimulate an exaggerated inflammatory response and contribute to the clinical syndrome [38]. Consistent with this hypothesis, levels of p21 were significantly higher in placentas from PE (Fig. 1, Fig. 4A), but again there were no significant differences in p16 or cGAMP (Fig. 1C). Sudan-Black-B staining was strong in the PE and IUGR placentas, predominantly in the syncytiotrophoblast where discrete foci could be identified (Fig. 3). Immunostaining revealed nuclear syncytial localisation of p21 (Fig. 4A) and modified histone, γH2AX (Fig. 4B) in the pathological placentas, providing evidence for DDR pathway activation in these placentas.

### Oxidative stress challenges can induce senescence and DNA damage in term placental explants *in vitro*

**Fig. 5:**
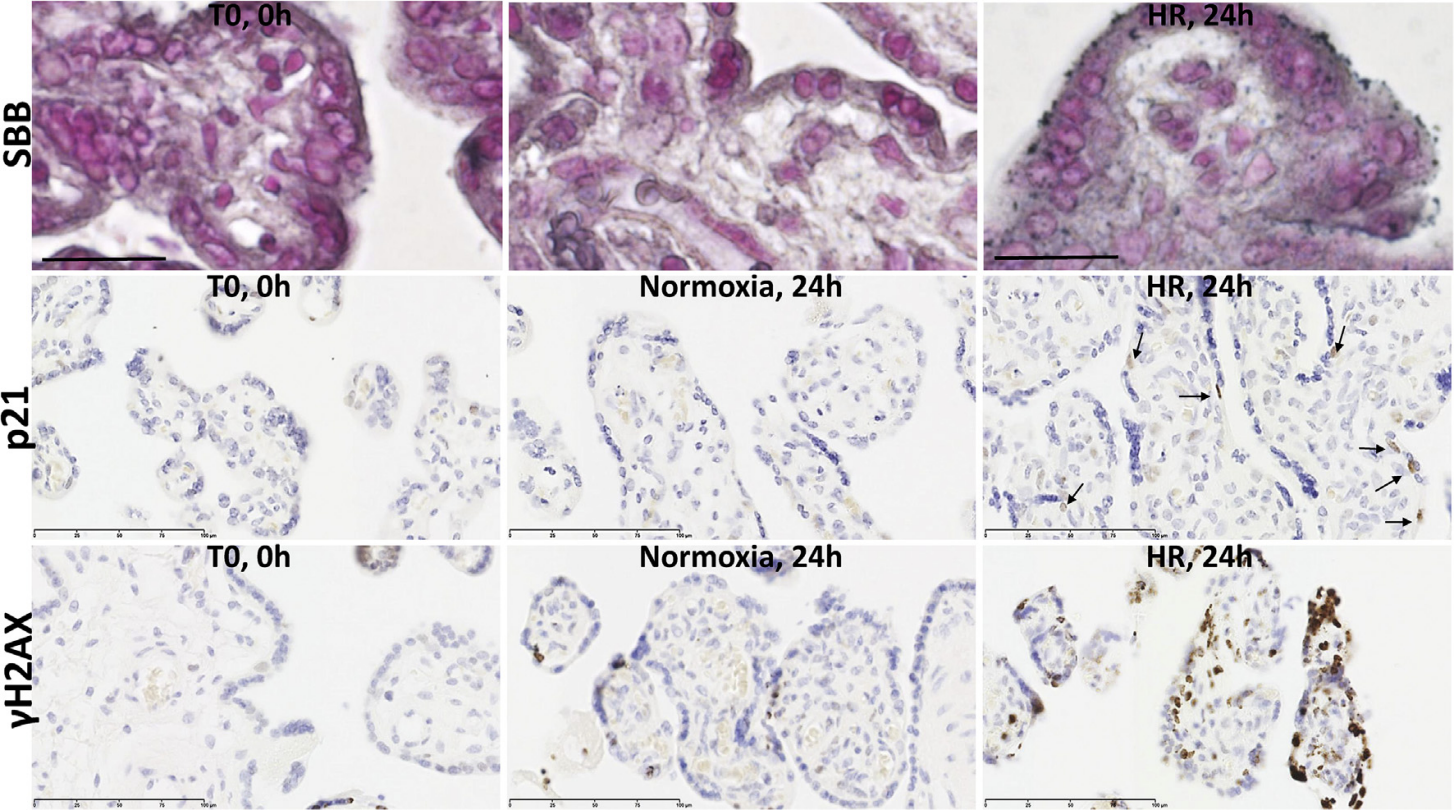
Evidence of senescence in placental explants challenged with oxidative stress of hypoxia-reoxygenation *in vitro*. Paraffin sections from term placental explants (N = 4), which were cultured under normoxia (N, 10% O2) or hypoxia-reoxygenation (HR, 1–10% O2) for 24 h were stained with the lipofuscin stain Sudan-Black-B (SBB; A), or immunostained with p21 (B) or modified histone, γH2AX (C), antibodies. Starting material (T0) was also stained to show the unstressed conditions at the start of the culture.

**Fig. 6:**
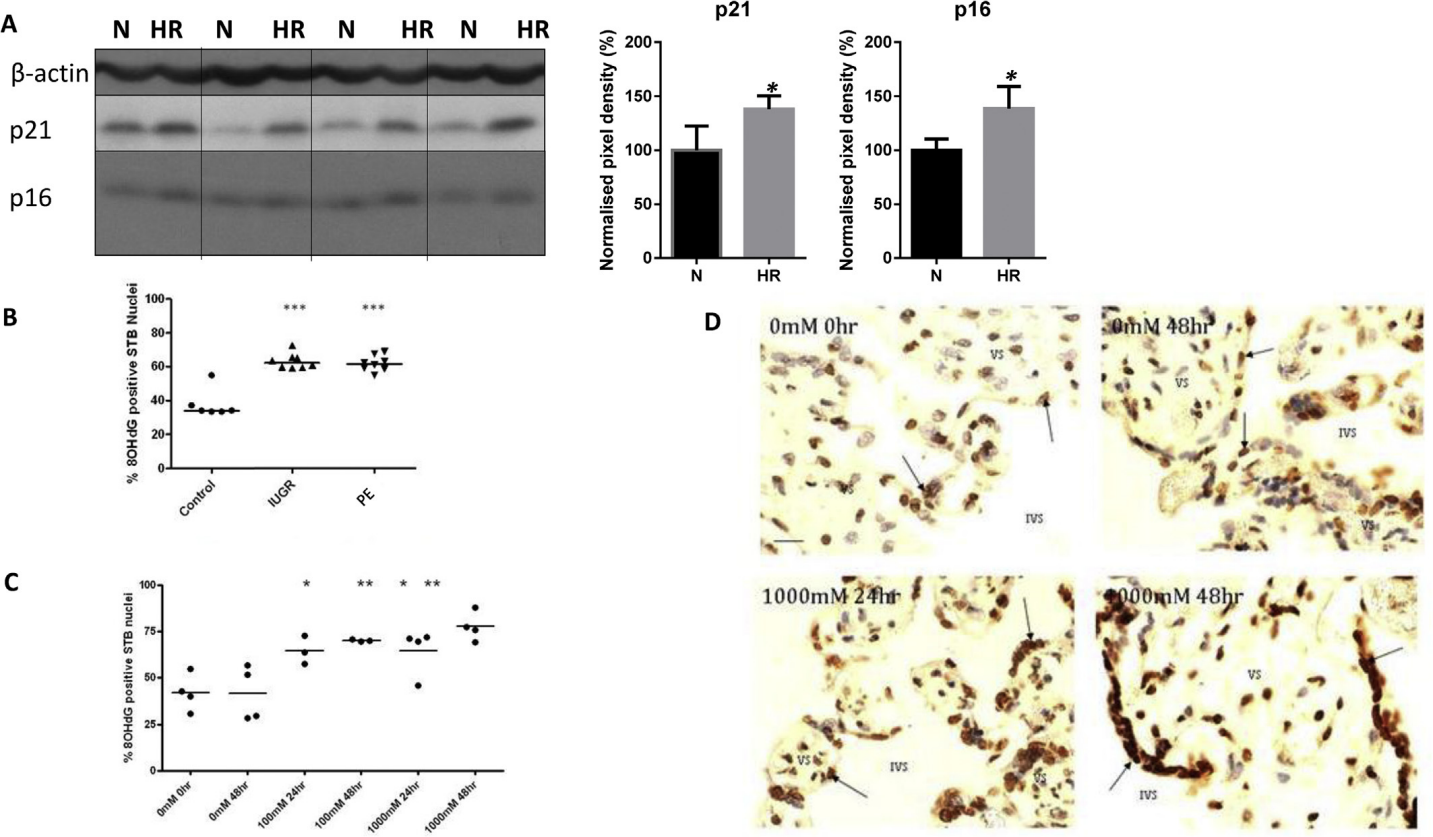
Evidence of senescence in placental explants challenged with oxidative stress of hypoxia-reoxygenation (A) or H_2_O_2_ (C–D) *in vitro*. A) Lysates from term placental explants (n = 4), which were cultured under normoxia (N, 10% O_2_) or hypoxia-reoxygenation (HR, 1–10% O_2_) for 20 h were immunoblotted with antibodies against p21 and p16. β-actin expression served to normalize gel loading. Normalized results (±SEM) are plotted, expressing normoxic controls as 100%. * Significant differences (P < 0.05, *t*-test). B-D) Immunoreactivity for 8-hydroxy-2′-deoxy-guanosine (8-OHdG), a marker of oxidised DNA, was examined in pathological placentas and in placental explants *in vitro*. B) Percentage of 8OHdG-positive syncytiotrophoblast nuclei was quantified in pathological and control placentas. Placentas from early onset IUGR (N = 5) and PE (N = 7) have increased proportions of 8OHdG-positive STB nuclei compared to gestational age-matched controls (pre-term controls, N = 5). Immunohistochemistry was used to detect 8OHdG-positive STB nuclei. Bar represents the group mean (Tukey's post hoc ***p < 0.0001). C-D) Quantification of 8OHdG-positive syncytiotrophoblast nuclei after H_2_O_2_ treatment (n = 3, 4). Term placental explants were cultured with 0–1000 mM H_2_O_2_ for 24–48 h, and fixed for immunohistochemical detection of 8OHdG. A higher proportion of 8OHdG-positive STB nuclei were observed in explants cultured in 1000 mM H_2_O_2_ when compared to control explants. Unpaired *t*-test between treatments showed statistically increased percentages of 8OHdG-STB in 100 mM and 1000 mM H_2_O_2_ after both 24 h culture, compared to the 0 mM 0hr control (*p < 0.05). There was an increase in the proportion of positive STB nuclei in 1000 mM after 48 h compared to the 0 mM 48hr control (**p < 0.001). (VS, villous stroma; IVS, intervillous space; arrows indicate positive STB nuclei; scale bar 20 μm).

Acute challenge (24 h) with HR induced similar changes in placental explants *in vitro* (Fig. 5, Fig. 6), with significant increases in the aggregation of lipofuscin, as detected by SBB (Fig. 5A), and significant increases in p21 (Fig. 5, Fig. 6A) and p16 (Fig. 6A), and increased nuclear foci of γH2AX (Fig. 5C) compared to normoxic controls.

Oxidative stress causes DNA damage, and we examined immunoreactivity for 8-hydroxy-2′-deoxy-guanosine (8-OHdG), a marker of oxidised DNA (Fig. 6B–D). The percentage of immunopositive nuclei was increased in pathological placentas compared to age-matched controls, with no differences between PE vs. IUGR (Fig. 6B). These changes could be mimicked *in vitro* in term placental explants challenged with H_2_O_2_ (0–1 M) for 24–48 h (Fig. 6C–D).

## Discussion

This study provides evidence of senescence in normal placentas across gestation, in post-mature placentas and in pathological pregnancies. We report significantly increased levels of p21, p16 and cGAMP in homogenates of healthy placentas with gestational age. This is consistent with a report showing p16, p21, p53 and SA-β-gal in term syncytiotrophoblast [6]. Londero et al. [29] found increased levels of 8-OHdG, APE1 and p53 with gestational age, but a reduction in p21. This difference may reflect the method of collection of early pregnancy samples. Our 7–17 week tissues were collected using chorionic villus sampling that avoids stress induced by suction curettage [39] that may raise levels artificially *ex vivo*. Placental ageing thus seems to be a regular response, and this physiological phenomenon was first described in the 1970s [[18], [19], [20]]. As pregnancy advances, placental cells undergo morphological changes, such as nuclear pyknosis [18], fibrinoid accumulation [19], and oxidative modification of nucleic acids [20], indicative of senescence.

Given the evidence of ageing in healthy placentas across gestation, we analysed a collection of archival paraffin and EM sections of post-mature placentas from patients who delivered 14–20 days after their due delivery dates in the 1970s. It is now recognised that the risk of fetal demise increases with gestational age in term patients, as there is a significant increase in the rate of stillbirth commencing at 36–37 wk gestation [22,23]. Consequently, such severe post-maturity is rarely seen in modern obstetric practice for delivery is usually induced after 41 wk. These severely post-mature placentas showed significant staining of the syncytiotrophoblast with Sudan-Black-B, a marker for aggregates of oxidised lipids, proteins and metals referred to as lipofuscin in ageing cells. SBB staining was largely absent in healthy term placentas. The post-mature placentas also showed increased nuclear abundance of p21 and staining intensity for cGAMP in the syncytiotrophoblast. The latter is indicative of increased cytoplasmic DNA, consistent with the abnormal nuclear appearances indicative of dissolution of the normal chromatin structure and potential release of DNA. Hence, our data show a striking increase in the incidence of senescence in the post-mature placentas (41–44 wk), compared to term controls (39–40 wk), validating the concept of physiological trophoblast ageing. Our results are in agreement with those of Maiti et al. who studied term placentas (39 wk), late term placentas (>41wk) and placentas from unexplained stillbirth. These authors reported increased aldehyde oxidase 1 expression, increased oxidation of DNA/RNA (8-OHdG) and lipids, perinuclear location of lysosomes, and larger autophagosomes in both later term and stillborn placentas, compared to placentas from term deliveries [21]. Overall, these findings suggest that premature ageing of the placenta could account for the placental dysfunction that characterizes some cases of stillbirth.

We next analysed evidence of senescence in pathological samples from early-onset PE (PE) and found significant increases in Sudan-Black-B staining, syncytiotrophoblast nuclear expression of p21 and nuclear foci containing modified chromatin (γH2AX) compared to term and pre-term controls. There were no significant differences in the levels of p16 or cGAMP among the groups. Placentas from cases of PE suffer more severe malperfusion than those from IUGR alone [37], which may stimulate an exaggerated inflammatory response that contributes to the clinical syndrome of PE [38]. Consistent with this hypothesis, levels of p21 were significantly higher in placentas from PE than in IUGR alone, but again there were no significant differences in p16 or cGAMP. Our data are supported by previous reports of telomere shortening in placentas from IUGR and PE pregnancies [28].

Hypoxia-reoxygenation is a potent inducer of oxidative stress, inflammation and apoptosis in term placental explants [34,40]. In this study, the HR challenge also induced senescent changes in term placental explants, which displayed significant increases in lipofuscin staining, p21 and p16, and nuclear foci of modified histone γH2AX, compared to normoxic controls. In addition, our *in vitro* experiments show that oxidative stress causes DNA damage. Compared to cultures under normoxia, treatment of term placental explants with H_2_O_2_ for 24 or 48 h induced an increased percentage of nuclei immunopositive for 8-hydroxy-2′-deoxy-guanosine (8-OHdG), a marker of oxidised DNA/RNA. Similar changes were observed in pathological placentas, consistent with the hypothesis that oxidative stress is the main inducer of senescence *in vivo*.

In conclusion, we show increasing levels of senescence in normal placentas with gestational age, and in pathological placentas. Oxidative stress triggers these changes in placental explants, and may be the precipitating insult *in vivo*. In severe cases, the consequent pro-inflammatory senescence-associated secretory phenotype may contribute to the pathophysiology of early-onset pre-eclampsia, for many of the cytokines released, such as IL-1, IL-6, IL-8, are common to both conditions.

## Conflict of interest

The authors have no conflict of interest to declare.

## Funding sources

Supported by the Wellcome Trust (084804/2/08/Z).
